# Using audit and feedback to guide tailored implementations of measurement-based care in community mental health: a multiple case study

**DOI:** 10.1186/s43058-023-00474-8

**Published:** 2023-08-14

**Authors:** Mira D. H. Snider, Meredith R. Boyd, Madison R. Walker, Byron J. Powell, Cara C. Lewis

**Affiliations:** 1 Department of Psychology, West Virginia University, 53 Campus Drive Morgantown, Morgantown, WV 26505 USA; 2 Department of Psychology, University of California Los Angeles, Los Angeles, USA; 3 Center for Health Equity Research, University of North Carolina, Chapel Hill, USA; 4 Center for Mental Health Services Research, Brown School, Washington University in St. Louis, St. Louis, USA; 5 Division of Infectious Diseases, John T. Milliken Department of Medicine, Washington University School of Medicine, Washington University in St. Louis, St. Louis, USA; 6 Center for Dissemination and Implementation, Institute for Public Health, Washington University in St. Louis, St. Louis, USA; 7 Kaiser Permanente Washington Health Research Institute, Seattle, USA

**Keywords:** Audit and feedback, Tailored implementation, Measurement-based care, Case study, Pattern analysis

## Abstract

**Background:**

Audit and feedback (A&F) is an implementation strategy that can facilitate implementation tailoring by identifying gaps between desired and actual clinical care. While there are several theory-based propositions on which A&F components lead to better implementation outcomes, many have not been empirically investigated, and there is limited guidance for stakeholders when applying A&F in practice. The current study aims to illustrate A&F procedures in six community mental health clinics, with an emphasis on reporting A&F components that are relevant to theories of how feedback elicits behavior change.

**Methods:**

Six clinics from a larger trial using a tailored approach to implement measurement-based care (MBC) were analyzed for feedback content, delivery mechanisms, barriers to feedback, and outcomes of feedback using archival data. Pattern analysis was conducted to examine relations between A&F components and changes in MBC use.

**Results:**

Several sites utilized both aggregate and individualized data summaries, and data accuracy concerns were common. Feedback cycles featuring individual-level clinician data, data relevant to MBC barriers, and information requested by data recipients were related to patterns of increased MBC use.

**Conclusions:**

These findings support extant theory, such as Feedback Intervention Theory. Mental health professionals wishing to apply A&F should consider establishing reciprocal feedback mechanisms on the quality and amount of data being received and adopting specific roles communicating and addressing data quality concerns.

**Trial registration:**

ClinicalTrials.gov Identifier: NCT02266134.

Contributions to the literature

- There are several components of audit and feedback interventions that are hypothesized to improve its impact on the uptake of new clinical behaviors. Individual-level feedback, feedback that was relevant to clinic-specific implementation barriers, and auditor responsiveness to data requests from clinics may increase use of measurement-based care in a tailored implementation initiative.
- Although implementation teams endorsed that data accuracy and flexibility to customize feedback was important to the audit and feedback process, these factors were not associated with changes in clinical behaviors.
- These findings highlight how specific feedback characteristics, timing of feedback, and organizational systems impact audit and feedback quality.

## Background

Despite a growing body of literature describing efforts to implement evidence-based practices (EBPs) to treat mental health disorders, the integration of EBPs into community mental health settings remains slow and challenging to sustain [2]. Tailored implementation approaches that account for determinants of behavior change while attempting to increase the use of a new clinical practice have outperformed standardized approaches to implementing EBPs [1]. Various approaches to tailoring exist, including adapting strategies to known determinants identified through previous experience or literature, conducting needs assessments to identify determinants and modify strategies pre-implementation, and iteratively adapting strategies throughout the implementation process in response to emergent determinants. It is unclear which tailoring approach is superior [29],however, the latter may be the most responsive to local contexts. Audit and feedback (A&F, the collection and delivery of performance data to clinicians to inform their clinical behaviors [30], is an implementation strategy that supports tailoring by establishing a feedback cycle through which tailoring decisions are evaluated and modified.

A&F is readily combined with other implementation strategies and has been applied to a range of healthcare settings, including cardiac rehabilitation [11], pain management [12], acute care [13], and the United States’ Veterans Affairs system [16]. A Cochrane review of 140 clinical trials indicated that A&F can increase the adoption of target behaviors by clinicians, particularly when there are discrepancies between baseline and desired performance [19]. A&F has also been linked to modest improvements in clinician behavior change [14] and increased EBP fidelity [34]. Despite the potential benefits of A&F, its effectiveness for changing clinician behaviors is equivocal [14, 19], and the specific mechanisms by which feedback influences target behaviors are not well understood [8]. Summaries of this literature have been limited by the heterogeneity of A&F interventions [24], as their structure (e.g., feedback specificity, methods for relaying feedback, frequency of delivery) is often driven by specific needs of clinicians and their contexts [10]. The Improved Clinical Effectiveness through Behavioural Research Group [17] indicated that variation in just five A&F components can yield as many as 288 unique approaches, making it difficult to draw conclusions about its utility [4].

Published A&F interventions are not consistently informed by relevant behavior change theories [5, 7, 9, 24], and descriptions of A&F in the literature lack sufficient detail to understand A&F component application. Optimal ways of applying theory-based recommendations for implementing A&F still need to be identified [5, 8, 15, 18]. Current recommendations posit that effective A&F is driven by the contents of feedback, the context in which feedback is received, recipient characteristics, and the nature of the target behavior [5, 8]. Thus, detailed information on how these components manifest in real-world applications of A&F is key to supporting future research [18].

The parent study of the current investigation compared tailored and standardized approaches to implementing measurement-based care (MBC) across twelve rural and urban community mental health centers in the Midwest [25]. MBC engages clinicians in routine assessment of patient outcomes during clinical encounters to guide decision-making [33]. The tailored arm of the parent study included A&F as a core implementation strategy to guide the selection and deployment of other strategies [26], providing a unique opportunity to examine A&F characteristics across multiple settings and relate the components of multiple A&F applications to feedback theory [5, 8]. The current study aimed to characterize team-based A&F across six community mental health centers by describing the specific feedback components, settings in which feedback occurred, delivery processes involved, and barriers to delivering feedback. Existing propositions from the A&F literature (i.e., teams perceived accuracy of audit data,individualized nature of data,relevance of data to identified barriers,engagement in modification of audit data,successful fulfillment of data tailoring requests) were also examined to determine the extent to which these elements were related to clinicians’ use of MBC.

The current study used data from a cluster randomized controlled trial comparing standardized and tailored approaches to implementing measurement-based care (MBC) for adults with depression across twelve rural and urban community mental health centers in the Midwest (see [25]). MBC engages clinicians in routine assessment of patient outcomes during clinical encounters to guide decision-making [33]. The standardized arm of the study was conceptualized as a “best practices” and “one-size fits all” approach to implementation, while the tailored arm represented a “collaborative and customizable” approach that sought to target clinic-specific barriers to implementation. The tailored arm of the parent study included A&F as a core implementation strategy to guide the selection and deployment of other strategies [26], providing a unique opportunity to examine A&F characteristics across multiple settings and relate the components of multiple A&F applications to feedback theory [5, 8]. Because A&F was part of the tailored approach, the current study focuses specifically on clinics randomized to that arm (for a full study description see [26]). The current study aimed to characterize team-based A&F across clinics by describing the specific feedback components, settings in which feedback occurred, delivery processes involved, and barriers to delivering feedback. Existing propositions from the A&F literature (i.e., teams perceived accuracy of audit data,individualized nature of data,relevance of data to identified barriers,engagement in modification of audit data,successful fulfillment of data tailoring requests) were also examined to determine the extent to which these elements were related to clinicians’ use of MBC.

## Methods

### Study context

Clinicians from a multi-site, not-for-profit community mental health organization participated in four-hour training sessions centered on evidence for and use of MBC. In these training sessions, clinicians learned to integrate the Patient Health Questionnaire 9-Item self-report measure (PHQ-9; Kroenke, [22]) in the treatment of adult clients (ages 18 and over) with depression. In the tailored arm, a group of clinicians, clinic managers, and office professionals participated in implementation team meetings every three weeks for five months of the “active implementation period” [25].

#### MBC guidelines

Each clinic in the parent study operated under a guideline for delivering MBC [23] that indicated the PHQ-9 should be administered, reviewed, and discussed with each adult client diagnosed with depression at every session. Teams in the tailored arm were invited to alter this guideline to fit the needs of their clinic (e.g., administering PHQ-9 s to every adult client).

#### Implementation team meetings

Implementation team meetings aimed to engage teams in identifying clinic-specific barriers to MBC implementation and selecting strategies to address those barriers. Although team activities varied by clinic, several components were consistent across clinics. First, teams had the option of reviewing data from a needs assessment regarding their clinic’s unique barriers and identifying barriers to prioritize. This needs assessment was informed by the Framework for Dissemination [28] and assessed contextual factors of influence across six domains: norms and attitudes,structure and process,resources; policies and incentives; networks and linkages; media and change agents [26]. Second, teams considered the option of tailoring the MBC guideline at their clinic. Third, teams received audit reports reporting on MBC implementation efforts. Finally, teams nominated members to serve as chair, secretary, and data expert. The chair created agendas and led meetings, the secretary took notes to ensure action step completion, and the data expert reviewed and presented the audit report to the team.

#### Audit and feedback

To support tailoring in team meetings, the auditor (MS) extracted PHQ-9 penetration and fidelity data (two important implementation outcomes that are indicators of behavior change; [27, 31] from the electronic health record (EHR) and compiled electronic audit reports. Penetration was defined as the number of individual sessions in which a PHQ-9 was completed out of all possible eligible sessions, and fidelity was indicated by whether PHQ-9 scores and graphs of PHQ-9 data were reviewed with clients. Audit reports were discussed in team meetings by all members to review MBC performance, ensure data accuracy, request adjustments to the audit report if needed (either adding to or altering data presented), and guide implementation decisions. As clinic-specific MBC guidelines were updated by the team, the clients reflected in the audit reports were adjusted accordingly. Team members had opportunities to pose questions to research staff concerning data collection to expand their understanding of the data.

### Design

The current study was conducted post-hoc, utilizing data from the parent study to examine characteristics of A&F at each clinic and rates of penetration during active implementation. A descriptive multiple-case study approach [36] was selected to describe A&F procedures at the individual clinic level (within-case) and comparatively (across-cases), allowing investigators to assess trends in the evolution of A&F characteristics across multiple clinics as well as associations between A&F characteristics and MBC penetration.

### Participants

Six implementation teams were formed across six community mental health clinics in Indiana and Tennessee. See Table 1 for a summary of clinic features. Team members were approached and consented for the parent study during an hour-long informational session that occurred 1 month prior to training. Participants were invited to join the implementation team based upon baseline scores on measures of social influence at the clinic (identified as an “opinion leader”; [35] and positive attitudes toward MBC (identified as a “champion” of MBC, [21]. Clinic managers and office professionals were also invited to join the team to ensure role representation. For a full description of the recruitment procedures, see Lewis et al. [26].

**Table 1:** Cross-case summaries

**Clinic descriptors**				**MBC guideline**			**A&F content and requests**		Extended reports?
Case	State	Urban	Size	Modified?	Decision meeting	Final guideline	Added A&F content	Other requests/impacts	
1	TN	No	Small	Yes	4th	First 10 new clients for each clinician over the age of 18 with a depression diagnosis	• MBC penetration reported separately for the following: ◦ Clients meeting criteria for modified MBC guideline ◦ All clients ages 18 and older with depression diagnoses (i.e., original MBC guideline)	• Requested the following changes to EHR: ◦ Indicator of MBC eligibility in client charts ◦ Program code for MBC ◦ Fidelity questions in individual progress notes	Yes
2	TN	Yes	Large	Yes	3rd	All clients ages 12 and older	• Volume of clinical services provided each month • MBC penetration data calculated for the following: ◦ Clients ages 12 + ◦ Clients ages 18 + ◦ Clients ages 21 + with intake in the past month • MBC fidelity question responses	• Requests for fidelity data supported the addition of a new “fidelity data report” to be embedded in the EHR that could be directly downloaded and viewed by clinic staff	Yes
3	IN	Yes	Small	No	N/a	All clients ages 18 and older with a depression diagnosis	• MBC penetration reported separately for the following: ◦ Clients on MBC guideline ◦ All clients ages 18 and older receiving individual therapy • Number of new intakes with an associated: ◦ Depression diagnosis ◦ PHQ-9 score ◦ Elevated PHQ-9 score • MBC fidelity question responses	• Requested a direct link to the PHQ-9 entry form to be added to individual therapy progress notes in the EHR	No
4	IN	Yes	Large	Yes	2nd	All clients ages 12 and older	• MBC penetration data at level of individual clinicians • MBC penetration data level of clinical teams (i.e., adult services, child services, intake)		Yes
5	IN	No	Medium	Yes	1st	All clients ages 12 and older	• MBC penetration data at level of individual clinicians • MBC penetration reported separately for the following: ◦ Clients on MBC guideline ◦ All clients ages 18 and older with depression diagnoses (i.e., original MBC guideline)	• Requested changes to individual therapy progress note format in the EHR such that MBC fidelity questions were more easily visible^a^ • Requested graphing function for PHQ-9A scores^a^	No
6	IN	Yes	Large	Yes	1st	Clients ages 12 and older who receive individual therapy during the first week of the month	• MBC penetration data at level of individual clinicians • MBC penetration for clinicians who may be recruited for additional training • MBC penetration data for 1^st^ week vs. entire month of services • MBC penetration for clients ages 12 and older with a depression diagnosis	• Requested adjustment to layout of PHQ-9 score graphs in EHR for easier printing and review	Yes

Clinic size based on number of clinicians employed at the time of assignment. Small < 15, medium 15–20, large > 20

MBC penetration = percentage of clients eligible per MBC guideline who received PHQ-9 in at least 80% of sessions; percentage of clinicians who delivered PHQ-9 to clients eligible for MBC guideline at least 80% of the time

MBC fidelity questions = (1) “Did you review the PHQ-9?” or (2) (if applicable) “Why didn’t you administer the PHQ-9?” and (3) “Did you review the PHQ-9 graph?”

Extended reports = implementation team requested audit reports to be delivered after the active implementation period

^a^A&F requests from implementation team not granted during active implementation period

Across participating clinics, 22 clinicians, 13 clinic managers, and five office professionals participated in at least one team meeting. Teams varied in size, ranging from four to nine members, typically reflective of clinic size. Meeting attendance ranged from 58 to 95% of members at each meeting. Throughout active implementation, five members left for other positions outside the clinic. Of these, four found other clinic staff to replace them on the team. Most team members were Caucasian (98%), identified as female (85%) and were an average age of 41.59 years old. Further, 70% were licensed clinicians and 52% supervised others. The teams included the study principal investigator (CCL), who served as team facilitator, and an auditor (MS) from the research team who worked for the organization’s research institute. The auditor collected raw data from the EHR and summarized these data into audit reports.

### Materials

#### Electronic health record reports

Self-report fidelity questions were added to individual therapy progress notes in the EHR at all clinics. EHR reports provided data on session-level PHQ-9 penetration and clinician answers to self-report fidelity questions. Fidelity questions included the following: (1) “Did you review the PHQ-9?” or (2) (if applicable) “Why didn’t you administer the PHQ-9?” and (3) “Did you review the PHQ-9 graph?”. These data were downloaded monthly by the auditor, de-identified, and added to the audit report.

#### Audit reports

Audit reports were created electronically and de-identified prior to delivery via encrypted email to team members. Specific contents and formats of the reports varied between meetings in response to data availability as well as team requests. For instance, teams may have requested to view penetration data for the entire clinic or at the individual clinician level; while other teams may not have been able to see clinician-level scores due to delays in updating certain reporting features within the EHR.

#### Auditor request log

The auditor maintained a request log to capture alterations to audit reports and additional data desired by teams. Items that were not completed were flagged, along with a brief rationale for why the task could not be completed on time or at all.

#### Implementation strategy tracking document

We tracked implementation strategy use in the context of the larger study; for a full description of our approach see Boyd, Powell, Endicott, and Lewis [3]. The tracking embraced recommendations for specifying and reporting implementation strategies proposed by Proctor et al. [32] including (1) naming and defining implementation strategies using existing taxonomies in the implementation literature and (2) operationalizing strategies by specifying the actor performing the strategy, action performed, target of the action, temporality, dose, outcome affected, and rationale for strategy selection. A member of the research team (MB) listened to each team meeting and extracted details consistent with the recommended operationalization, including direct quotations from team members and the auditor during data review. For the current study, only mentions of A&F were extracted to describe the A&F procedures and related actions that occurred in team meetings (e.g., changes in data available through the EHR reports, requests made by the team for additions and deletions, when updates were actually made to audit reports by the auditor).

### Procedures

#### Data collection

De-identified EHR reports were used to verify changes in PHQ-9 penetration and fidelity over time. Data were also obtained from implementation team audit reports and the auditor’s request log. Type of data present (e.g., individual clinician summaries versus clinic-level summaries) and changes in content or format of the report were extracted from each audit report. The implementation strategy tracking confirmed when the team discussed audit data, when the team discussed data validity concerns, types and frequency of audit requests made, and whether previous data requests were fulfilled.

#### Triangulation and data analysis

Variables from each data source were combined into a single data record and reviewed by three research team members (MB, MS, and MW) to avoid unbalanced attention toward any one data source. For example, notes from the auditor log and strategy tracking log were used in combination to confirm quotes from teams that they found data to be reliable at a given meeting. The combined data was used to create individual case and cross-case summaries [36]. Triangulated data informed chronological descriptions of each clinic’s A&F procedures. Case summaries emphasized A&F modifications from planned procedures, how teams used audit reports, and barriers to A&F. A cross-case summary of A&F procedures is provided in Table 1.

Pattern matching (Campbell, [6]), a qualitative case study approach that plots change in a measured outcome and assesses conditions coinciding with changes in the outcome trajectory, was used to assess relations between A&F characteristics (i.e., propositions of change) and changes in MBC penetration over time. Thirty theory-based A&F propositions were selected a priori from a published compilation [8], from which 20 testable propositions were retained after excluding those that did not vary across clinics or could not be examined in more than one case. A list of five propositions was selected with input from the team facilitator (CCL). See Table 2 for the final list of propositions and data sources for each.

**Table 2:** A&F propositions, data sources used to code propositions, and pattern matching results

Proposition		Data source			Pattern matching results					
No	Description	Audit report	Strategy tracking	Auditor log	Case 1	Case 2	Case 3	Case 4	Case 5	Case 6
1	MBC penetration will increase when implementation team members believe that audit reports accurately represent their clinical performance		X	X	50%	33%	50%	17%	43%	33%
2	MBC penetration will increase when audit reports include information that is relevant to MBC barriers identified by implementation team members during team meetings	X	X		83% ^b^	33%	83%^b^	17%	43%	33%
3	MBC penetration will increase when the audit report includes data that is reported at the individual clinician level	X			83% ^b^	67%^a^	83%^b^	67%^a^	29%	83%^b^
4	MBC penetration will increase when implementation team members make intentional adjustments to the design, format, or contents of the audit reports	X	X	X	50%	50%	50%	67%^a^	29%	33%
5	MBC penetration will increase when requests to adjust the design, format, or content audit reports are successfully accommodated by researchers or other clinic staff	X	X	X	67%^a^	17%	67%^a^	83%^b^	43%	33%

Audit report = data for confirming proposition was collected from audit report records delivered to implementation teams

Strategy tracking = data for confirming proposition was collected from the strategy tracking document

Auditor log = data for confirming proposition was collected from the auditor request log

Pattern matching results = percentage of cases in which satisfied propositions were followed by increased MBC penetration scores

^a^Satisfied propositions were followed by increased penetration in over 50% of implementation team meetings

^b^Satisfied propositions were followed by increased penetration in over 80% of implementation team meetings

Three investigators (MS, MW, MB) coded five A&F propositions as being satisfied (e.g., the audit report contained data at the level of individual clinicians) or not at each team meeting using triangulated data. Investigators compared proposition satisfaction codes (Y/N) for each clinic with monthly MBC penetration scores and the proportion of meetings where satisfied propositions were followed by increased MBC penetration were recorded. If penetration increased following the satisfaction of a proposition in over 50% of meetings, the proposition was considered “supported” at that clinic. In cross-case analyses, propositions being supported at more than one clinic indicated “replication.”

## Results

An overview of changes made by teams to MBC guidelines, A&F modifications, and additional requests from teams across cases are summarized in Table 1.

### Case 1 (cohort 1)

#### A&F summary

Audit reports summarized aggregated penetration for the entire clinic. Penetration scores included the following: the percentage of clinicians administering a PHQ-9 in at least one therapy session with an eligible client, the percentage of clinicians administering a PHQ-9 in at least 80% of sessions with clients meeting the MBC guideline, the percentage of eligible clients receiving at least one PHQ-9 during their sessions, and the percentage of eligible clients receiving a PHQ-9 in at least 80% of sessions. At the second meeting, clinic staff on the team received a list of services with clients with a depression diagnosis and associated PHQ-9 scores. This list identified clients for whom PHQ-9 data was not entered into the health record and verified the validity of the audit report (i.e., determined whether scores were missing due to noncompletion of the PHQ-9 or to clinician failure to enter PHQ-9 data). Four cycles of A&F (i.e., four meetings) were completed before team members endorsed accurate report data. No data expert was nominated; team members reviewed audit reports jointly. The most prevalent barrier was perceived report inaccuracy, attributed to errors in the programming developed to extract PHQ-9 penetration data from the EHR and miscommunication between the team and auditor regarding the locations and services types desired for the report. The auditor received data for building reports indirectly via a quality management employee of the community mental health organization, which increased burden to build reports in a timely manner.

Team members at this clinic advocated for modifications to the EHR that supported A&F across the organization. These included the following: an MBC guideline indicator in client charts to increase their visibility as an MBC target, a new program code that clients meeting MBC guideline criteria could be assigned to, and addition of fidelity questions to progress notes. These additions remained in the EHR long-term and supported MBC in future cases. Report inaccuracy in early meetings increased involvement of the organization’s quality management division in A&F report creation and delivery, and the auditor began attending meetings regularly in this and future cases to identify sources of inaccuracies and generate solutions. The auditor communicated requested changes to quality management and monitored submitted requests.

### Case 2 (cohort 2)

#### A&F summary

Audit reports included the same aggregate clinic-level data delivered to case 1. As in case 1, the auditor obtained penetration data through a quality management employee, hindering timely report completion such that the data expert did not receive, interpret, and share a summary as planned on at least two occasions. During these delays, data was delivered to the entire team 1 day or less before meetings. Team members expressed concern that the penetration scores were underreported and not all data were captured. To address this, the auditor shared an individual service data report modeled after the one used in case 1 with the case 2 data expert to confirm that all desired clinicians were included in the report.

### Case 3 (cohort 2)

#### A&F summary

Less than 6 months prior to active implementation at this clinic, a new EHR was implemented in this state’s branch of the organization which demanded new procedures for obtaining service data. An advantage of the new EHR was granting the auditor direct access to reports and eliminating the third party of the quality management team. All reports for this case contained the same aggregate data as previous cases. Uniquely, these reports specified the number of clinicians, clients, and services reflected in each penetration percentage score. Fewer than the expected number of clients at this clinic were recorded in the EHR as having a depression diagnosis. Like cases 1 and 2, fidelity data availability was delayed. Differences in how MBC guideline markers appeared in the new EHR also affected report-building. This new EHR did not provide a program code to quickly filter MBC candidate clients. As a result, the auditor required two separate reports (one with service records that included a PHQ-9 score, and one with a list of clients meeting the MBC guideline) followed by manual cross-checking. Modifications to audit reports at this site, namely, to include the number of intake sessions in which a PHQ-9 was delivered and/or a depression diagnosis was made, were a means of identifying the reason for low rates of service to clients who met the MBC guideline.

#### Feedback utilization

The A&F process at this clinic revealed that clinicians were less likely to diagnose depression in clients, even as a secondary or tertiary diagnosis. In response to learning that MBC penetration across the clinic was low, team members requested that individual service data be delivered to specific clinicians who might be more likely to interact with clients meeting criteria for the MBC guideline. The auditor delivered service reports to these clinicians outside of regularly scheduled team meetings, which included diagnoses of clients at the time of service and whether a PHQ-9 was administered.

### Case 4 (cohort 3)

#### A&F summary

Initial audit reports at this clinic included aggregated penetration and fidelity data. Penetration was reported for all clients who met the adapted MBC guideline and clients with any depression diagnosis. Continuing the format from case 3 reports, the auditor reported the number of clinicians, clients, and services reflected in each penetration score. Team members reported some data inaccuracy in early meetings. Upon review of data, team members stated that they believed a higher percentage of services should have associated PHQ-9 scores than was reported.

#### Feedback utilization

Team members reviewed service data between meetings after identifying that penetration was lower than expected. These data identified sessions in which clinicians were not entering PHQ-9 scores in the EHR and surfaced errors in EHR data extraction programming. The auditor facilitated communication between team members and quality management to ensure that these discrepancies were documented and addressed. Penetration scores from the audit reports were used to hold a clinic-wide competition incentivizing MBC across clinical teams. Prizes were awarded to clinicians with the highest penetration score in the fifth A&F cycle. Penetration data was also used to identify clinicians not using the PHQ-9 that might benefit from further support or training.

### Case 5 (cohort 3)

#### A&F summary

Aggregate penetration and fidelity data were included in every report and the auditor continued reporting the number of clinicians, clients, and services reflected in each score. As in previous cases, clinicians verbally endorsed higher rates of penetration than shown by reports. Clinicians regularly using the PHQ-9 for adolescents (PHQ-9A) at this clinic indicated concern that the order of the questions in the PHQ-9A entry form was inconsistent with the order in the adult form, increasing confusion during data entry and potentially affecting scores. The team requested changes to the EHR to improve usability of the progress note fidelity questions and PHQ-9 graphs. These requests were not immediately granted after being identified by quality management as “low priority.”

#### Feedback utilization

In response to concerns that penetration rates were underreported, the auditor sent individual service data to clinicians to rule out EHR programming errors or problems with timely entry of scores. The auditor identified that PHQ-9 scores entered into the EHR would not appear in the data if entered less than 24 h before the time of data extraction.

### Case 6 (cohort 4)

#### A&F summary

Every report delivered to this clinic contained aggregate penetration and fidelity data, as well as the number of clinicians, clients, and services represented in penetration scores. The team requested that all reports include individual clinician penetration for services with all clients 12 and older, even though this did not match formal changes to the MBC guideline to maximize MBC use in the first week of each month. To reflect this goal, aggregated penetration data were reported for both the first week of the month and the entire reporting period. Claims that reports were invalid were rarer at this clinic compared to previous cases. When this clinic entered active implementation, new features had been added to the EHR that streamlined report building by adding MBC guideline markers to raw data. In the fifth meeting, the team requested to expand the scope of reports to include clinicians outside of the implementation team. Penetration data for these clinicians was used to identify participants for a second MBC training held by existing champions during active implementation.

### Pattern analyses

Pattern analysis findings for each case are summarized in Table 2. Proposition 1 (i.e., team members perceived audit report data as accurate) was not followed by increases in MBC penetration scores more than 50% of the time for any case. Proposition 2 (i.e., audit report contains data that is pertinent to team-endorsed implementation barriers) was followed by increased MBC penetration more than 50% of the time for cases 1 and 3. Proposition 3 (i.e., audit report included data at individual clinician level) was followed by MBC penetration increases more than 50% of the time in all cases except case 5. Proposition 4 (i.e., team members request specific alterations to the A&F process) was only followed by improved MBC penetration for case 4 more than half of the time. Finally, proposition 5 (i.e., requested changes to the A&F process were successfully fulfilled by the auditor and incorporated into feedback) were followed by increased penetration more than half of the time for cases 1, 3, and 4.

In summary, three of the five tested propositions corresponded with an increase in MBC penetration more than half of the time and were replicated across more than one clinic. These supported propositions included the following: (1) including data in the audit report that was directly relevant to team-based implementation goals, (2) including data that reflects individual clinician performance, and (3) auditor follow-through on data modification requests.

## Discussion

This study reported the proceedings of six clinics’ use of A&F for increasing PHQ-9 utilization in community mental health settings. This study applied a cross-case pattern analysis to test whether changes in the means by which A&F components were delivered co-occurred with changes in MBC use by sites in an active implementation period.

Content changes of audit reports included new aggregated penetration categories, breakdowns of penetration scores by individual clinician, and the addition of fidelity data. Several modifications were requested to address clinic-specific barriers, such as identifying clinicians or services in which PHQ-9 s were not being administered as intended. Three teams influenced A&F procedures beyond their own clinic by requesting changes to processes through which reports were made (e.g., adding questions to progress notes in the EHR), and the auditor was leveraged as a resource to influence MBC information technology procedures beyond the scope of A&F (e.g., adding links to PHQ-9 in the progress note, editing PHQ-9 symptom trajectory graphs).

A&F procedures in the current study represented an iterative process that fostered collaboration between team members, research personnel, and the organization’s quality management team. In five of six clinics, evolution was observed in audit report contents and structure. Changes in the means by which data was made accessible to the auditor (e.g., new reporting features in the EHR) occurred as result of joint problem-solving between the auditor and team members. Several A&F components that occurred consistently across clinics reflect “best practices” in the extant literature [4, 20], including the use of repeated feedback cycles, feedback representing recent performance of a target behavior, individualized feedback, and multimodal presentations of feedback (e.g., giving documents to team members and reviewing data orally in meeting). These core features of our A&F may have affected MBC penetration by increasing tailoring opportunities and discussion around perceived MBC obstacles. Many clinics requested monthly audit reports into the sustainment period, which can be interpreted as evidence that reports were helpful to teams. The only site that did not request additional reports (case 3) was the clinic that received the least amount of actionable data and had less experiencing tackling A&F barriers due to very few candidate clients being identified.

Data validity concerns were a common barrier across clinics. Manual report-building by the auditor introduced human error into the development of reports, and the accuracy of penetration summaries also depended upon timely entry of PHQ-9 scores by clinicians. This difficulty emphasizes the importance of EHR functionality that includes the flexibility to generate and automate service-delivery reports. However, it may be argued that the manual creation of reports and verbal delivery of feedback had the benefit of establishing an ongoing relationship between the auditor and feedback recipients. Recipient trust of data sources has been implicated as an important factor in designing A&F interventions [5, 8, 18]. It is possible that increased clinician engagement in the development of feedback could increase acceptance of report contents. In the current study, this relationship was leveraged to tackle tasks outside the scope of planned A&F procedures (e.g., adding the PHQ-9A to the EHR). Data availability also changed across clinics and over time. Events like the implementation of a new EHR in some clinics had a clear impact on the availability of data needed to create reports. Clinics beginning MBC implementation after the EHR implementation had better data access because quality management had sufficient time to establish or modify PHQ-9 and fidelity reporting features.

Pattern matching analyses revealed that individualized data was associated with penetration improvements in five clinics. This finding is consistent with literature positing that feedback is more actionable to its recipients when it reflects relevant clinicians’ individual performance [8, 20]. One explanation for the utility of individualized data could be that it activates social comparisons between feedback recipients and increases social demands to engage in MBC. Additionally, individualized data is arguably more useful because it allows recipients to directly observe their own performance gaps or identify individuals in their organization that would benefit from additional support [5, 8].

The fulfillment of data requests made by teams was also associated with improved penetration in three clinics, while the act of making requests alone was only associated with improved penetration at one clinic. This finding emphasizes the importance of establishing trust between feedback systems and recipients in which recipients can rely on the feedback cycle to be appropriately *responsive* to their needs [8].

Providing data that mapped onto specific MBC barriers, such as narrowing the focus of data to specific types of clinical encounters like intakes appointments with fewer associated PHQ-9 records, was associated with improved penetration in two clinics. Feedback containing barrier-relevant information may be important because it enables stakeholders to modify interventions based on more relevant determinants of practice [1],however, this feedback component was limited by the auditor not taking a full spectrum of potential barriers into account proactively. Rather, barriers were addressed in the reports if explicitly requested by team members and provided in the form requested by members (e.g., aggregated vs. individual level data).

Finally, teams perceiving reports as accurate was not related to increased penetration. Notably, our data did not reflect a direct assessment of team members’ perceived report accuracy. Given that perceptions were coded from transcribed discussions in meetings as well as the types of team requests made, it is possible that patterns identified in the current investigation to not fully map onto the attitudes of individual team members. Additionally, perceived mismatch between lived experiences of clinicians and the data they are viewing may undermine progress toward penetration when clinicians are not adequately challenged or engaged by the data. Having accurate data and perceiving legitimate discrepancies between actual and desired performance may also have been undermined by the absence of other essential A&F components, such as individual clinician representation or actionability. It is important to acknowledge that multiple mechanisms of feedback are expected to work in combination (e.g., credibility, social influence, actionability) to affect feedback cycles [5, 8]. Furthermore, it is important to keep in mind that each site created unique MBC guidelines, so effectiveness of various A&F characteristics in these pattern analyses may have been impacted by the diverse nature of the target behaviors being recorded across teams.

## Strengths and limitations

The current study was strengthened by its multiple case approach, which allowed an investigation of A&F trends across clinics. Case summaries were strengthened by focusing on theory-relevant characteristics of A&F and providing detailed descriptions of procedures to inform implementation in similar contexts. Limitations of the current study included its non-experimental design, post hoc data collection, and generalizability, given that clinics reflected only one parent organization. Additionally, the current study focused on audit-specific influences on the implementation of MBC and does not reflect interactions between A&F and external influences, such as organizational culture and climate, availability of resources, and workforce capacity issues.

## Conclusion

Future research should include experimental investigations that compare methods of applying A&F and directly test feedback mechanisms. A&F characteristics with promising support in the reported cases include providing individualized data at the level of clinicians, opportunities for dialogue around feedback, and feedback that is relevant to clinic-specific implementation barriers. These findings support extant theory in A&F, such as Feedback Intervention Theory [5]. Mental health professionals who wish to apply A&F in their practice should consider mechanisms through which they can establish reciprocal feedback on the quality and amount of data being received as well as adopting specific roles communicating and addressing data quality concerns.

## Data Availability

Not applicable.

## Abbreviations

EBP: Evidence-based practices
A&F: Audit and feedback
MBC: Measurement-based care
EHR: Electronic health record
PHQ-9: Patient Health Questionnaire, Short Form
